# The significance of intra-abdominal pressure in neurosurgery and neurological diseases: a narrative review and a conceptual proposal

**DOI:** 10.1007/s00701-019-03868-7

**Published:** 2019-03-25

**Authors:** Paul R. A. M. Depauw, Rob J. M. Groen, Johannes Van Loon, Wilco C. Peul, Manu L. N. G. Malbrain, Jan J. De Waele

**Affiliations:** 1 Department of Neurosurgery, Elisabeth Tweesteden Hospital (ETZ), Tilburg, The Netherlands; 2 Elisabeth Tweesteden Hospital, Hilvarenbeekseweg 60, 5022 GC Tilburg, The Netherlands; 3 Department of Neurosurgery, University Medical Center Groningen, University of Groningen, Groningen, The Netherlands; 4 Department of Neurosurgery, University Hospitals Leuven, KU Leuven, Leuven, Belgium; 5 Department of Neurosurgery, Leiden University Medical Center (LUMC) and The Hague Medical Center (HMC+), Leiden, The Netherlands; 6 Intensive Care Unit, University Hospital Brussels (UZB), Jette, Belgium; 7 Faculty of Medicine and Pharmacy, Vrije Universiteit Brussel (VUB), Brussels, Belgium; 8 Department of Critical Care Medicine, Ghent University Hospital, Ghent, Belgium

**Keywords:** Vertebral venous system, VVS, Intra-abdominal pressure, IAP, Intra-abdominal hypertension, IAH, Prone position, Idiopathic intracranial hypertension, IIH, Hydrocephalus, Traumatic brain injury, TBI, Prone position, Cerebrospinal venous system, CSVS, Extradural neural axis compartment, EDNAC

## Abstract

Intra-abdominal pressure (IAP) is a physiological parameter that has gained considerable attention during the last few decades. The incidence of complications arising from increased IAP, known as intra-abdominal hypertension (IAH) or abdominal compartment syndrome in critically ill patients, is high and its impact is significant. The effects of IAP in neurological conditions and during surgical procedures are largely unexplored. IAP also appears to be relevant during neurosurgical procedures (spine and brain) in the prone position, and in selected cases, IAH may affect cerebrospinal fluid drainage after a ventriculoperitoneal shunt operation. Furthermore, raised IAP is one of the contributors to intracranial hypertension in patients with morbid obesity. In traumatic brain injury, case reports described how abdominal decompression lowers intracerebral pressure. The anatomical substrate for transmission of the IAP to the brain and venous system of the spine is the extradural neural axis compartment; the first reports of this phenomenon can be found in anatomical studies of the sixteenth century. In this review, we summarize the available knowledge on how IAP impacts the cerebrospinal venous system and the jugular venous system via two pathways, and we discuss the implications for neurosurgical procedures as well as the relevance of IAH in neurological disorders.

## Introduction

Increased intra-abdominal pressure (IAP) is recognized as a significant contributor to organ dysfunction in many critically ill patients. Although it primarily has been studied in surgical ICU patients suffering from abdominal catastrophes such as abdominal trauma, peritonitis, and pancreatitis, it has also been associated with massive fluid resuscitation.

IAP is defined as the steady-state pressure within the abdominal cavity. Under physiological conditions, values of up to 5 mmHg are considered normal in adults [14]. IAP varies inversely with intrathoracic pressure during normal breathing, while positive pressure ventilation affects IAP directly [57]. Valsalva manoeuvers, such as coughing, sneezing, squatting, or singing loudly, increase IAP dramatically for short periods of time [12]. In conditions such as obesity or pregnancy, basic IAP may range from 10 to 15 mmHg [18, 39, 40].

Intra-abdominal hypertension (IAH) is defined as a sustained IAP elevation equal to or above 12 mmHg, usually documented in three consecutive measurements taken at 4- to 6-h intervals. Since 2004, the World Society of the Abdominal Compartment Syndrome (WSACS, recently renamed Abdominal Compartment Society) has developed a cohesive approach to the management of IAH and ACS, fostered education and research, and developed consensus statements and definitions [20, 35]. Recently the society changed its name to the Abdominal Compartment Society [Ki.]

While the impact of elevated IAP has been described anecdotally in neurological conditions, the anatomy and pathophysiology of the related vascular structures have not yet been described in detail. In this manuscript, we will focus on both issues and review the literature on the relevance and impact of elevated IAP in neurological and neurosurgical practice.

## Methods: search strategy and paper selection

Relevant articles were identified by searching PubMed from 1918 to 2018 and also by screening the references of the identified articles.

The following search items were used: “Intra-abdominal pressure AND neurosurgery,” “intra-abdominal pressure AND spine surgery,” “intra-abdominal pressure AND TBI,” “intra-abdominal pressure AND hydrocephalus,” “intra-abdominal pressure AND IIH”.

After the search by the first author, all literature was reviewed by the coauthors and supplemented with relevant articles. The coauthors are experts in various areas related to IAP: history, neuroanatomy, intensive care medicine, and neurosurgery.

## Historical perspective

The relationship between the IAP and the intracranial pressure (ICP) has been suspected for more than 100 years.

The existence of both the cranial venous system and the vertebral venous system (VVS) was already known in the sixteenth century, but it was Breschet in 1828–1832 who published the first detailed drawings, accurately visualizing the multiple anastomoses c.q. the anatomical connections between the intracranial venous system and the VVS.

**Fig. 1:**
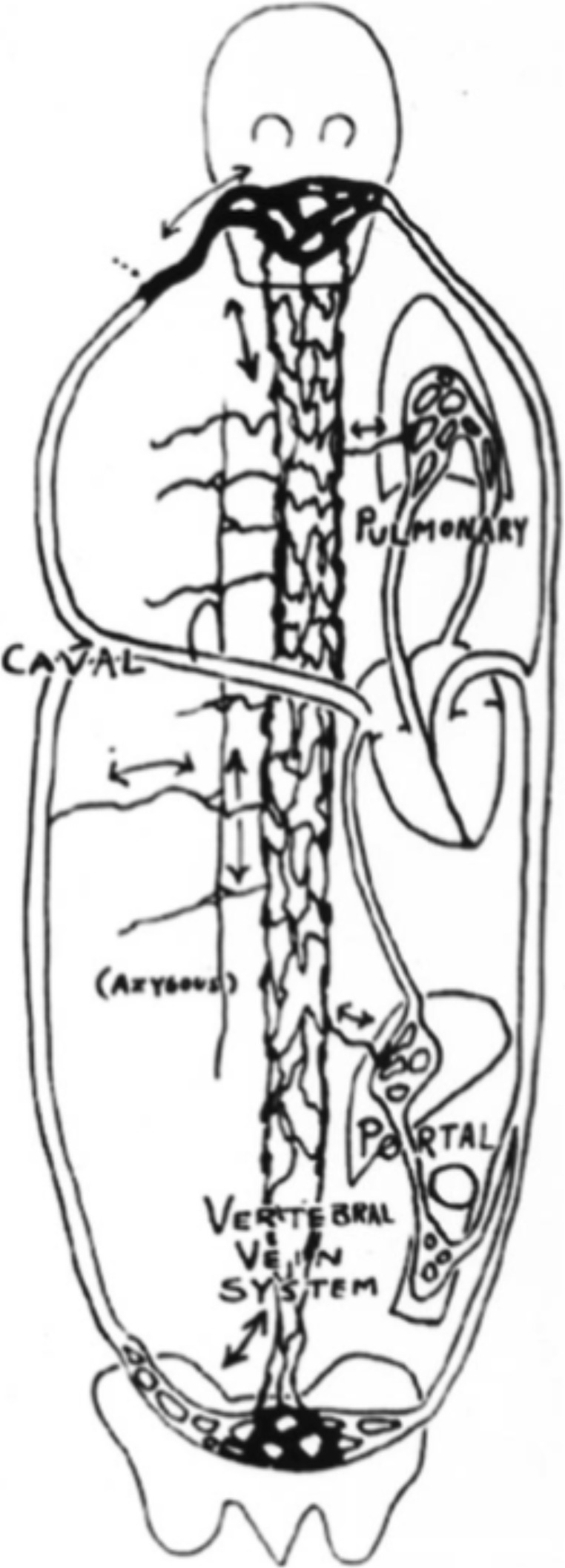
Batson’s 1940 drawing of the valveless pathways within and about the vertebral column

In 1940, the first of Batson’s two landmark articles appeared, dealing with properties of the spinal veins. His radiological study provided an explanation for the spread of metastasis and infections through these vertebral veins, into the spine and the central nervous system [4], while bypassing both the liver and lungs [25]. Batson’s drawing in 1957 illustrates the extent of the connections of the multiple veins of the valveless spinal epidural venous network. These veins are now known as Batson’s veins (Fig. 1). His original paper from 1940 still is the most frequently cited in spinal oncology [16].

**Fig. 2:**
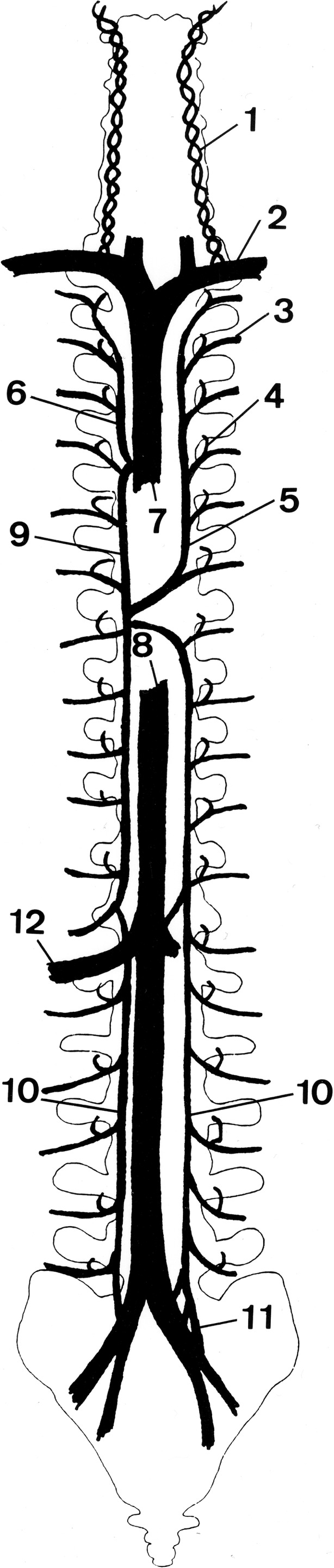
Schematic representation of the connections of the vertebral venous system (VVS) with the vena cava system showing the vertebral vein (1), subclavian vein (2), segmental thoracic (intercostal) and lumbar veins (3), intervertebral vein (4), hemiazygos vein (5), internal thoracic vein (6), superior vena cava (7), inferior vena cava (8), azygos vein (9), ascending lumbar vein (10), sacral venous plexus (11), and the renal vein (12). Reproduced from Groen et al. [62] with permission from Lippincott Williams & Wilkins

Multiple anatomical studies over the past century have shown that the cranial and vertebral venous system has connections to both the deep systemic, valved venous system, including the inferior and superior vena cava, to the valveless superficial veins in the face, head, and back and to the thoracoabdominal wall [4, 26, 30] (Fig. 2).

**Fig. 3:**
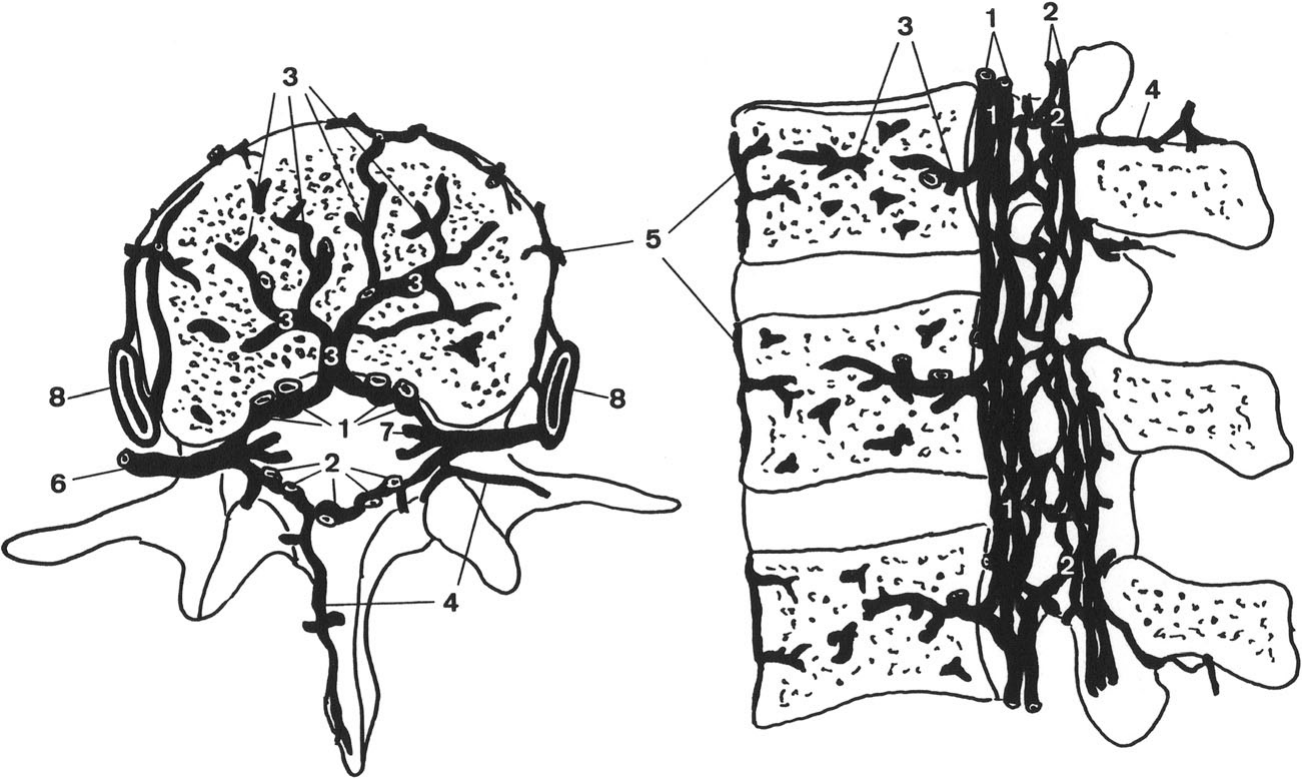
Schematic representation of the vertebral venous system (VVS) at the lumbar area showing the anterior internal vertebral venous plexus (1), posterior internal vertebral venous plexus (2), basivertebral veins (3), posterior external vertebral venous plexus (4), anterior external vertebral venous plexus (5), intervertebral vein (6), radicular vein (7), and the ascending lumbar vein (8). Reproduced from Groen et al. [62] with permission from Lippincott Williams & Wilkins

In 2000, Dwight Parkinson described the extradural neural axis compartment (EDNAC), which is characterized by valveless veins that allow blood to run freely in either direction between the orbit and the os coccygis [52]. Later, in 2006, Tobinick et al. introduced the term cerebrospinal venous system (CSVS), to emphasize the continuity/connectivity of the intracranial and the spinal venous networks. The first of the two main parts of this network are the intracranial veins. The second part consists of the vertebral venous system (Fig. 3) which courses along the entire length of the spine. The intracranial veins richly anastomose with the VVS in the suboccipital region, especially the suboccipital sinus. Caudally, the CSVS freely communicates with the sacral and pelvic veins and the prostatic venous plexus [48, 59]. However, we will continue to use the term EDNAC, since in recent literature, this was the first accurate description of this venous network.

### Anatomical considerations and pathophysiology

The pivotal system responsible for the regulation of pressure within the spinal canal and partially within the intradural compartment seems to be the venous structures belonging to the extradural neural axis compartment. The vertebral venous system is a part of that structure and parallels, joins, and at the same time bypasses the longitudinal veins of the thoracoabdominal cavity. The VVS is connected with both the superior and the inferior vena cava by means of numerous connections with the intrathoracic (hemi)azygos venous system and the intra-abdominal lumbar veins. Also, numerous connections exist with the subcutaneous veins of the chest and the abdomen, the vertebral veins of the neck, and the sacral venous plexus.

Since the VVS is valveless, flow and the direction of flow depend (1) on pressure gradients between the IAP, the intrathoracic pressure (ITP) and the pressure in the spinal canal which is largely determined by the ICP; and (2) on hydrostatic factors and gravitation forces that may occur with changing of posture or position (lying, sitting, standing).

Morphological features that seem of the utmost importance are the fact (1) that the VVS has a large volume (probably 20 times larger than that of the contributing arteries [10]); (2) that it has numerous valveless connections with the cranial, spinal, thoracic, abdominal, and subcutaneous veins; and (3) that a significant part of this system is situated inside the rigid spinal canal (mass effect if congestions occurs [29, 50]).

Two important mechanisms are involved in the interaction between the abdominal compartment and the central nervous system. In the first mechanism, the VVS is the anatomical substrate for transmission of the IAP to the spinal canal and the cranium.

This valveless VVS, which allows bidirectional flow, is a huge reservoir for blood. Pressure change in one compartment of the human body and can thus shift venous blood to the other compartment. Epstein and colleagues described this as a large-capacity “venous lake,” out of which blood may flow into the brain and spinal canal and into which blood may flow from the brain, depending on variations in posture and thoracic pressure or IAP [26].

**Fig. 4:**
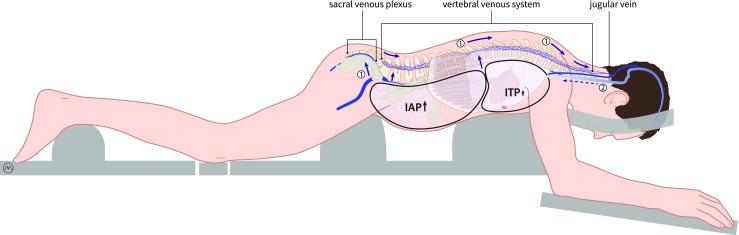
Schematic drawing illustrating the concept of the two pathways. In the first pathway, an increase in IAP can cause backflow through the sacral venous plexus and the vertebral venous into the spinal canal. This can cause congestion of venous blood in the spinal canal and can cause flow of venous blood into the brain. In the second pathway, an increase in IAP can cause an increase in ITP (intrathoracic pressure) which in turn results in a back pressure on the jugular veins and decreases the drainage of the CSF and the venous blood. Drawing made by Medical Visuals in collaboration with Dr. Paul Depauw

Secondly, an increased IAP has an influence on the intrathoracic pressure (ITP). Cerebrospinal fluid (CSF) and the brain’s venous drainage both leave the brain via the jugular veins and the VVS. Elevations in the IAP are transferred into the thoracic compartment, which in turn results in a back pressure on the jugular veins and decreases the drainage of the CSF and blood, leading to an increased ICP [56, 43] (Fig. 4).

Interaction between the abdomen and the central nervous system has been studied in humans and in animal models [17, 21]. The Monro-Kellie hypothesis states that the cranium is a rigid vault that contains brain tissue, CSF, and blood [61]. If one of the three components increases in size, the volume of the other two has to decrease, in order to maintain equilibrium and to prevent a raise of ICP. An increase in IAP (acute or chronic) will result in a subsequent increase of the intracranial volume of venous blood and can cause an increased ICP.

The relationship between IAP and ICP has not only been demonstrated by the study of Batson in 1940 but also by more recent and more modern techniques. As the optic nerve sheath (ONS) is part of the dura mater and the optic nerve is surrounded by CSF, a change in pressure within the subarachnoid space is detected by ultrasonography. An analysis of prospectively collected data of patients who underwent laparoscopic procedures showed that an acute elevation in IAP significantly increased the optic nerve sheath diameter (ONSD). The changes in the ONSD reflect a temporary and reversible increase in ICP [23]. Recent literature shows an increase in the number of publications reporting total perioperative visual loss (POVL) in healthy patients after elective laparoscopic surgery [53].

## IAP measurement technique

The intravesicular IAP measurement is convenient and is considered the gold standard [46, 58]. IAP is the pressure concealed within the abdominal cavity as defined superiorly by the diaphragm, anteriorly and posteriorly by the abdominal wall, and inferiorly by the pelvic floor. In general, the abdominal compartment transduces pressure evenly throughout the cavity. IAP can thus be measured via every cavity within this defined region (intra-gastric, inferior vena cava, rectal, vaginal). Femoral vein pressure cannot be used as a surrogate measure of IAP [15].

Conditions for a reproducible intravesicular IAP measurement are measured at end-expiration; performed in the supine position; zeroed at the iliac crest in the mid-axillary line, using a priming volume in the bladder of < 25 mL of saline; measured 30–60 s after instillation to allow for bladder detrusor muscle; and measured in the absence of active abdominal muscle contractions. Values need to be expressed in millimeters of mercury (1 mmHg = 1.36 cmH_2_O). In a prospective comparative study, Al-Abassi et al. showed that intra-bladder pressure measurement is a simple, minimally invasive method that may reliably estimate the IAP in patients placed in supine position [2].

Body position can affect IAP. In a multicenter analysis of different body positions, Cheatham et al. found that mean IAP values were significantly different at each head of bed position [7]. Even the position of the transducer located in the mid-axillary line or located at the level of the symphysis pubis can cause a bias [19].

Both of these studies emphasize the importance of a standardized approach to IAP measurement to ensure the reproducibility of the results.

In the future, new techniques may become available using cost-effective solid-state pressure transducers, inserted via the bladder or stomach or by ingestion of wireless capsules, allowing continuous monitoring of IAP [58].

## IAP and neurosurgical procedures

In neurosurgical operating procedures, the influence of posture on ICP is well-known. In the early days, when surgery of the nervous system was developing, one of the main factors that complicated craniotomy was the inability to control ICP. Brain prolapse and uncontrollable swelling in awake patients was the reason that brain surgery results were disappointing, and in the absence of anesthesiology support, the associated surgical mortality was frustratingly high, especially in posterior fossa surgery [6].

### IAP and spine surgery or posterior fossa surgery: the prone position

Nowadays, with excellent anesthesiologic support and sophisticated neurosurgical equipment, intracranial and spinal surgery has become safe and controllable. However, correct positioning of the patient is key for a smooth and uncomplicated operative procedure, especially with respect to cerebral venous drainage. With a patient in prone position (for spine surgery or posterior fossa surgery), a free-hanging abdomen is mandatory to prevent venous congestion of the operative field [1, 8]. Prior to surgery, it is verified manually whether the abdomen is free-hanging via “palpation of the belly to exclude any external pressure.” In order to secure an uncompromised venous return, the use of thoracolumbar supports has become standard practice in neurosurgery (Fig. 4). Although not generally accepted in the intensive care (ICU), these supports also can be beneficial for lowering IAP in adult patients with respiratory distress syndrome (ARDS) who require ventilation in the prone position [3, 36, 45, 54].

One of the implications of inadequate positioning during spine surgery in the prone position is the increased blood loss associated with the higher IAP values that were described in some studies. A rise in IAP can cause venous congestion in the pelvis and abdomen. This may lead to backflow of venous blood into large valveless VVS, which can result in venous congestion in the rigid spinal canal, which makes spinal surgery complicated [8, 51] (Fig. 4).

The study by Han et al. was the first prospective study to show the effect of BMI on IAP and intra-operative blood loss during spinal surgery. Besides this cumbersome blood loss, the visual operating field is obscured, making microsurgery a more difficult and frequently a higher complexity procedure because of the risk of damaging neurological structures and creating temporary or permanent postoperative loss of function.

**Fig. 5:**
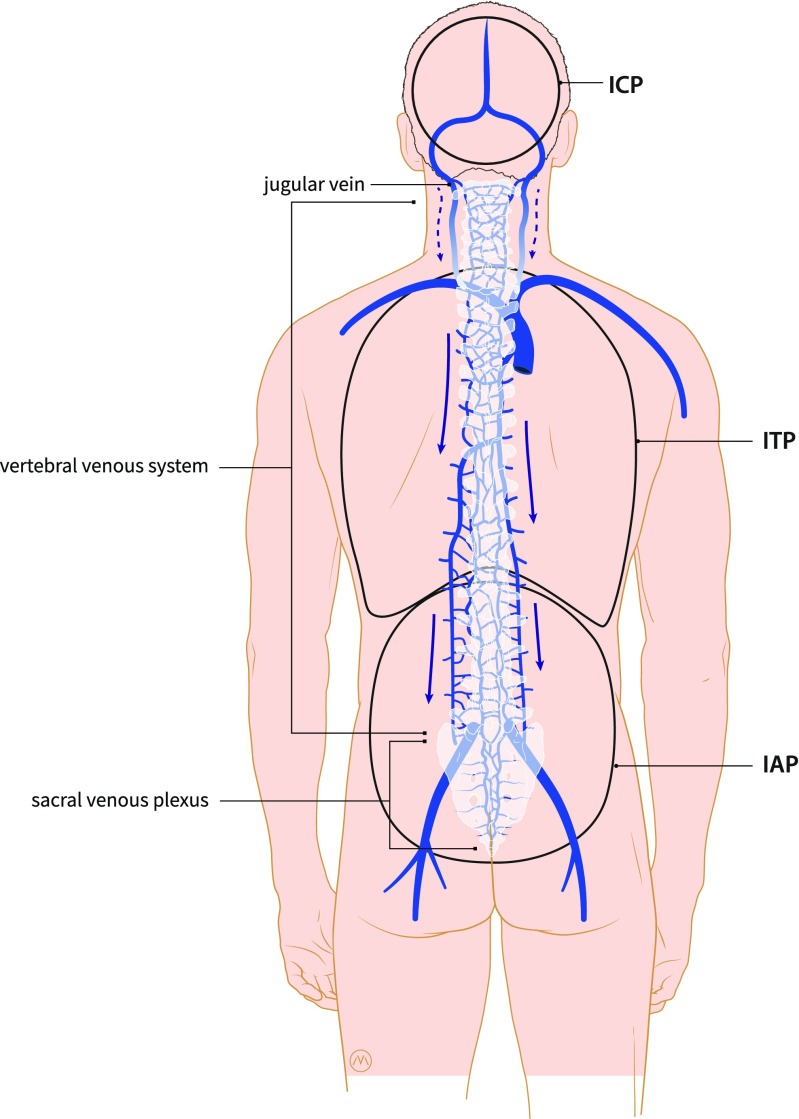
Schematic drawing illustrating the preferred venous outflow of the brain in the upright position and the possible influence of the IAP and ITP on this flow. The blue arrow shows the preferred outflow in the valveless vertebral venous system (VVS). The dashed arrow shows the reduced flow through the jugular venous system in the upright position. Drawing made by Medical Visuals in collaboration with Dr. Paul Depauw

Another consequence of inadequate positioning can be brain swelling during posterior fossa surgery or supratentorial craniotomy in the prone position. As mentioned earlier, raised IAP causes a rise of the intrathoracic pressure. This causes an increase in back pressure in the jugular veins and decreased drainage of venous blood and CSF (Fig. 5).

However, it is clearly not yet common knowledge that venous congestion in the spinal canal is a problem in surgery in prone position and that this problem involves the IAP. In a review by Chui et al. about the prevention of complications related to prone positioning during spinal surgery, the reviewers did not consider the importance of a free abdomen to reduce the incidence of postoperative visual loss was not considered [8, 22].

### The effect of IAH on internal shunting of CSF in case of hydrocephalus

In the neurosurgical community, IAP is regarded as a negligible variable when considering the use of a ventriculoperitoneal shunt for treating hydrocephalus. It is a general misconception in the neurosurgical community that IAP is around 0 mmHg and therefore does not interfere with CSF drainage into the peritoneal cavity.

A study performed by Sahuquillo et al. on 60 patients requiring a ventriculoperitoneal shunt evaluated the relationship between body mass index (BMI) and IAP and provided data to estimate IAP in order to help neurosurgeons with the selection of the appropriate shunt. IAP was measured through an intraperitoneal catheter during shunt surgery. The authors concluded that in obese and overweight patients, neurosurgeons should take IAP into account when selecting both the most adequate differential pressure valve and the distal cavity to place the distal catheter. IAH could be a reason to choose another distal cavity, for example the pleural cavity [11, 55].

Therefore, in the event of neurologic deterioration in patients with a pre-existing ventriculoperitoneal shunt during critical illness, IAP should be monitored and IAH should be treated promptly. IAP should also be monitored in critically ill, sedated, and mechanically ventilated patients with a ventriculoperitoneal shunt, since neurologic examination is unreliable in these circumstances.

Raised IAP due to constipation, ileus, or small-bowel obstruction has also been reported to play a role in malfunctioning ventriculoperitoneal shunts in patients with hydrocephalus [44].

## IAP in neurological conditions: the effect of the IAP on the ICP

### IAP and TBI

Various case reports have described raised ICP following abdominal closure after laparotomy in polytrauma patient. A patient whose acute subdural hematoma was removed in combination with a splenectomy for trauma-developed acute bleeding from his right transverse sinus and brain herniation as the abdomen was closed, indicating a rise in venous pressure [31].

The first well-conducted clinical study evaluating the relationship between IAP and ICP in 15 patients with traumatic brain injury was published by Citerio et al. in 2001 [9]. They conducted a prospective non-randomized observational study systematically measuring the effect of increased IAP, by positioning a soft 15-L bag of water on the patient’s abdomen. Obviously, patients could only be included in this study after the acute phase of their injury, when no intracranial hypertension was present. The authors found that placing a weight on the patient’s abdomen generated a significant increase in IAP and a concomitant and rapid increase in ICP, central venous pressure (CVP), and jugular bulb pressure (IJP). All these values reached a plateau within seconds and remained increased until weight removal.

A number of publications reported decompressive laparotomy (DL) as a last resort to decrease otherwise untreatable intracranial hypertension after TBI. In one series, abdominal fascia release resulted in the decrease of ICP in 11 out of 17 patients. All 11 patients survived [34].

In a study by Neville et al., 15 patients with normal opening pressures on lumbar puncture and referred for CSF analysis were asked to artificially increase their IAP through a Valsalva maneuver [49]. All were able to elevate their CSF pressure to values above 25 cmH_2_O, and one patient even achieved a value of 47 cmH_2_O. The authors were prompted to perform this study after the observation that patients were referred for fundoscopy due to increased CSF pressure while they had no symptoms of intracranial hypertension.

In a series of 102 patients who sustained blunt brain injury, Scalea et al. evaluated the serial application of decompressive craniectomy (DC) and decompressive laparotomy (DL) to treat multicompartment syndrome (MCS) and to lower ICP; 24% of the patients required both interventions. Fifteen patients had DC before DL and nine had DL before DC. The mean time between DC and DL was 3.4 ± 6 days. Mean ICP decreased significantly after both DC and DL (*p* < 0.05) [56].

In a recent review, Lauerman et al. described the pathophysiology of a multicompartment syndrome (MCS) and current treatment considerations for patients with TBI given the effect of MCS. In MCS, intracranial, intrathoracic, and intra-abdominal compartment pressures are interrelated. The authors concluded that TBI care should include ICP control as well as minimization of intrathoracic and intra-abdominal pressure as far as clinically possible [38].

Another indication of the link between cerebral venous outflow and ICP is the beneficial effect on a raised ICP in TBI that is achieved by elevating the head of the bed [56]. Another important (iatrogenic) factor in TBI is fluid management. For instance, fluid therapy to support cerebral perfusion pressure (CPP) may cause retroperitoneal and visceral edema, increasing IAP, which in turn can increase ICP. Ventilatory manoeuvers for treating respiratory failure (e.g., recruitment) may increase intrathoracic pressure, limiting venous return, and thus increasing ICP and decreasing CPP [56]. All these findings support the claim that a more profound understanding and appreciation of the role of the venous system in neurocritical care is vital [61].

### IAP and IIH

Idiopathic intracranial hypertension (IIH) is characterized by an elevation of intracranial pressure (ICP). In the primary form, there is no identifiable cause. IIH is a disorder that mainly affects obese women of childbearing age. Its prevalence has been estimated to lie between 0.5 and 2 per 100,000 of the general population. Clinical symptoms include headache; visual disturbances due to papilloedema and, less frequently, olfactory dysfunction; uni- or bilateral pulsatile tinnitus; diplopia due to sixth nerve palsy; and cognitive dysfunction [32]. Obesity is a consistent risk factor for the development of IIH [13, 33, 40]. Despite the association between IIH and an obese phenotype, the pathological mechanisms tying the two together are unclear. IIH is a rare disorder, while obesity is common [47].

The jugular venous system is commonly recognized as the main route of venous efflux from the brain. However, the jugular venous system has important flow limitations with changes in posture as described earlier in the pathophysiology [28]. A volume capacity of up to 1000 mL has been calculated for the extra-jugular venous system, which could be sufficient to take over the entire venous drainage of the brain [24, 26]. In 1970, Epstein and colleagues provided scientific evidence to support this hypothesis by demonstrating that when the sagittal sinus was injected with contrast media in rhesus monkeys in the upright position, the vertebral venous system was the main route of venous efflux from the brain, and the venous vertebral system continued to be an important venous outflow tract even in the supine position. They suggested that the vertebral venous plexus may act as a siphon, facilitating the flow of blood across the brain in the upright position [26].

Other studies confirmed that the anastomoses between the cranial and vertebral portions of the CSVS appear to serve an important function in providing pressure homeostasis to the intracranial venous system with changes in posture [27, 28, 60]. A systematic ultrasound analysis by Doepp described the variations of the cerebral venous drainage in the horizontal body position in healthy subjects and found the expected predominant jugular drainage in 72%. However, in 22%, the jugular equaled the extra-jugular drainage, and in 6%, the extra-jugular drainage was the major path of cerebral venous outflow [24].

Besides the anatomical variations of cerebral venous drainage, there is also a variation in abdominal compliance [5]. Abdominal compliance is defined as a measure of the ease of abdominal expansion, which is determined by the elasticity of the abdominal wall and the diaphragm [37, 42]. In case of a low abdominal compliance, a slight increase in intra-abdominal volume (and thus in IAP) could have a greater impact on ICP [41].

The balance between cerebral blood inflow and outflow is vital in maintaining normal ICP. Restrictions in venous outflow, for example due to a raised IAP, can be as important as mass accumulation within the cranium, if not more so [61] (Fig. 5).

The increased IAP in obese patients can thus influence ICP via the two pathways described in this literature review. The relationship between obesity and the onset of IIH is unclear for many reasons: first, there are many anatomical variants of venous drainage; second, there are differences in abdominal compliance between patients; and last, it is unclear by which pathway IAP has the most influence on ICP [61].

## Conclusions

The IAP is an important physiological parameter in neurosurgery and neurology. It has a close relationship with the ICP, and current evidence supports the concept that there are two pathways by which the IAP can be transmitted to the central nervous system. One pathway is by backflow through the venous plexus of the spinal canal and the intracranial veins. This valveless venous plexus provides a direct anatomic route from the pelvis to the eyes and the brain, and vice versa. This is a route with numerous anastomoses to the systemic venous circulation, including the venous circulation of the lungs, the renal veins, and the veins of the breasts. The second pathway is a direct effect via an increase in IAP, which causes a cranial excursion of the diaphragm. The elevated intrathoracic pressure and augmented central venous pressure causes a decrease in venous drainage from the central nervous system via the jugular system.

Current evidence suggests that increased IAP may play an important role during neurosurgical procedures in patients suffering from IIH or TBI and during hydrocephalus therapy. IAP measurement could provide relevant information to improve the safety of surgical procedures in spine surgery and posterior fossa surgery. Furthermore, it could optimize the treatment for IIH, TBI, and hydrocephalus. However, more prospective research in this field is needed.
